# Challenges in Modelling Hypoglycaemia-Associated Autonomic Failure: A Review of Human and Animal Studies

**DOI:** 10.1155/2016/9801640

**Published:** 2016-10-23

**Authors:** Manjula Senthilkumaran, Xin-Fu Zhou, Larisa Bobrovskaya

**Affiliations:** School of Pharmacy and Medical Sciences, Sansom Institute for Health Research, University of South Australia, Adelaide, SA 5000, Australia

## Abstract

Recurrent insulin-induced hypoglycaemia is a major limitation to insulin treatment in diabetes patients leading to a condition called hypoglycaemia-associated autonomic failure (HAAF). HAAF is characterised by reduced sympathoadrenal response to subsequent hypoglycaemia thereby predisposing the patients to severe hypoglycaemia that can lead to coma or even death. Despite several attempts being made, the mechanism of HAAF is yet to be clearly established. In order for the mechanism of HAAF to be elucidated, establishing a human/animal model of the phenomenon is the foremost requirement. Several research groups have attempted to reproduce the phenomenon in diabetic and nondiabetic humans and rodents and reported variable results. The success of the phenomenon is marked by a significant reduction in plasma adrenaline response to subsequent hypoglycaemic episode relative to that of the antecedent hypoglycaemic episode. A number of factors such as the insulin dosage, route of administration, fasting conditions, blood sampling methods and analyses, depth, duration, and number of antecedent hypoglycaemic episodes can impact the successful reproduction of the phenomenon and thus have to be carefully considered while developing the protocol. In this review, we have outlined the protocols followed by different research groups to reproduce the phenomenon in diabetic and nondiabetic humans and rodents including our own observations in rats and discussed the factors that have to be given careful consideration in reproducing the phenomenon successfully.

## 1. Introduction

Hypoglycaemia is an unavoidable side effect of insulin therapy in both type 1 and advanced type 2 diabetes and can be life threatening. In type 1 diabetes patients, 2–4% of deaths have been attributed to hypoglycaemia [1]. Importantly, hypoglycaemic episodes in diabetic patients are not benign; they reduce the physiological defence against subsequent hypoglycaemic episodes leading to a condition called hypoglycaemia-associated autonomic failure (HAAF). The mechanisms that reduce the physiological response to hypoglycaemia are not fully understood; therefore, further research is needed into the possible mechanisms of HAAF to alleviate the suffering of diabetic patients. In order to achieve this, HAAF models (human and animal) have been developed. This manuscript reviews human and animal models of HAAF, describes different protocols used by researchers to establish the HAAF model, and highlights the challenges that we have experienced in our own laboratory while establishing the HAAF model in rats. Before we discuss the current models of HAAF, we will briefly describe the counterregulatory and symptomatic responses to hypoglycaemia in nondiabetic individuals and how these are altered in HAAF.

## 2. Counterregulatory and Symptomatic Responses to Hypoglycaemia 

**Figure 1:**
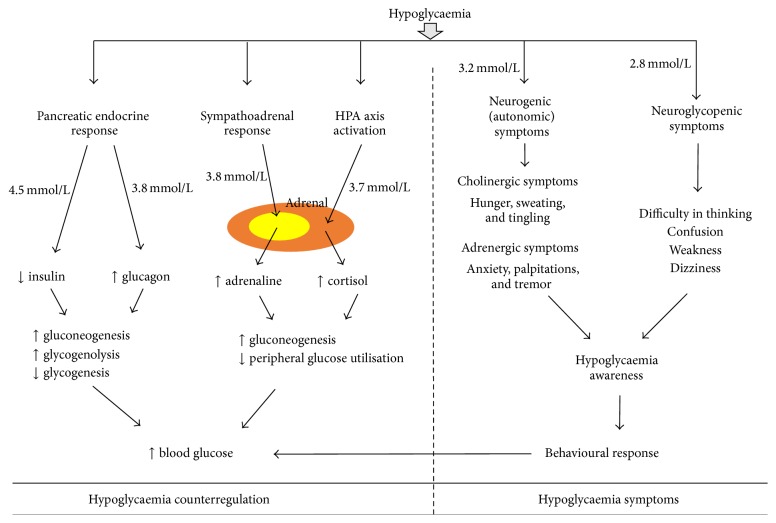
Counterregulatory and symptomatic responses to hypoglycaemia in nondiabetic humans. HPA axis: hypothalamic-pituitary-adrenal axis.

In nondiabetic individuals, falling plasma glucose concentrations trigger a characteristically organised sequence of responses to prevent further progression of hypoglycaemia (Figure 1) (for comprehensive review refer to [2, 3]). When the blood glucose falls below 4.5 mmol/L, the first line of defence is reduction in pancreatic insulin secretion; if this measure is insufficient and a further fall in blood glucose occurs below 3.8 mmol/L, the release of glucagon from the pancreas is triggered [4–7]. Glucagon restores blood glucose mainly by stimulating gluconeogenesis and glycogenolysis and reducing glycogenesis and glycolysis. It also increases the uptake of amino acids in the liver and mobilises glycerol from adipose tissue to be used for gluconeogenesis [8]. Glucagon release is also accompanied by activation of the sympathoadrenal system leading to the release of adrenaline from the adrenal gland. Adrenaline raises plasma glucose concentrations through an array of mechanisms including directly stimulating hepatic (and renal) glucose production, limiting glucose clearance by insulin sensitive tissues, mobilising gluconeogenic substrates such as lactate and amino acids from muscle and glycerol from fat, increasing glucagon secretion, and limiting insulin secretion [5, 9–11]. In addition to these, when the blood glucose falls further below 3.7 mmol/L activation of the hypothalamic-pituitary axis leads to the release of growth hormone and cortisol that further initiate the adaptive responses to hypoglycaemia by stimulating lipolysis, ketogenesis, and gluconeogenesis. A further reduction in plasma glucose levels below 3.2 mmol/L and 2.8 mmol/L leads to the generation of neurogenic symptoms such as palpitations, anxiety, tremor, hunger, sweating, and tingling and neuroglycopenic symptoms such as difficulty in thinking, confusion, weakness, and dizziness, respectively, which prompt the individual to ingest carbohydrates [12]. The neurogenic symptoms are reported to be largely due to sympathoneural rather than adrenomedullary responses as these responses are intact in bilaterally adrenalectomised individuals [13]. Palpitations, anxiety, and tremor are adrenergic symptoms caused mainly by noradrenaline released from sympathetic nerves, whereas hunger, sweating, and tingling are caused by acetylcholine released from sympathetic nerves. The neuroglycopenic symptoms result from glucose deprivation in the brain. It should be emphasised that severe hypoglycaemia in nondiabetic individuals is not physiological and should be considered as a hypoglycaemic disorder; such disorder can result from usage of drugs, hormonal disturbances (e.g., hyperinsulinism due to insulinoma), or critical illness [1].

## 3. Hypoglycaemia-Associated Autonomic Failure (HAAF)

**Figure 2:**
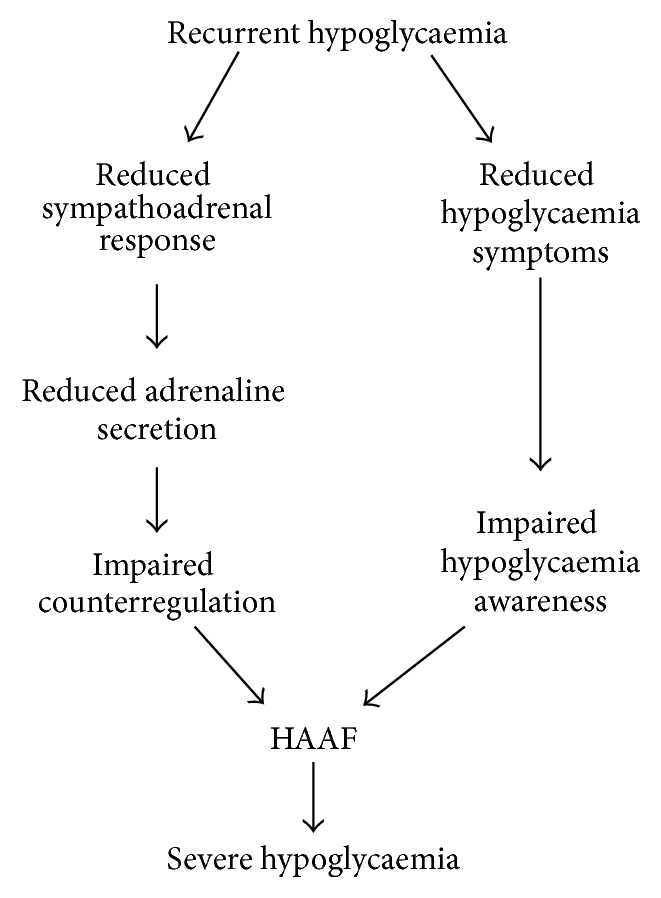
Recurrent hypoglycaemia leads to impaired adrenomedullary adrenaline response and reduced hypoglycaemia symptoms giving rise to the phenomenon of HAAF, which predisposes an individual to become more vulnerable to severe hypoglycaemia.

Most of the abovementioned counterregulatory and symptomatic responses against developing hypoglycaemia are typically compromised in people with type 1 diabetes and advanced type 2 diabetes [49]. The glucose levels at which these responses occur are referred to as glycaemic threshold which can be reset to higher glucose levels following chronic hyperglycaemia or to lower glucose levels following repeated hypoglycaemia [6]. In both type 1 and advanced type 2 diabetic individuals, insulin responses are absent and their glucagon levels fail to increase in response to hypoglycaemia [1, 50, 51]. In this setting, the sympathoadrenal response becomes critical in diabetic patients. However, repeated episodes of hypoglycaemia lead to the resetting of the threshold for the activation of the counterregulatory and symptomatic responses to a lower glucose level. Cryer has defined this phenomenon as hypoglycaemia-associated autonomic failure (HAAF) [10, 52]. The concept of HAAF postulates that in type 1 diabetes and advanced type 2 diabetes recent antecedent iatrogenic hypoglycaemia causes defective adrenomedullary adrenaline response and impaired awareness of hypoglycaemia (Figure 2). It should be emphasised that the HAAF phenomenon is distinct from the classic diabetic autonomic neuropathy, a common complication of diabetes [53]. Here we base our review on Cryer's definition of the phenomenon.

Defective counterregulation and impaired awareness of hypoglycaemia occur as the recent antecedent hypoglycaemia reduces the sympathoadrenal response and neurogenic and neuroglycopenic symptoms to subsequent hypoglycaemia [14, 19, 20]. Type 1 diabetes patients with defective glucagon and adrenaline response to hypoglycaemia have a 25-fold or greater increased risk of iatrogenic hypoglycaemia during intensive insulin therapy [54]. As the duration of diabetes increases, protection from hypoglycaemia, dependent upon sympathoadrenal activation and the release of circulating adrenaline, diminishes. The mechanisms of progressive sympathoadrenal impairment to hypoglycaemia in long duration diabetes are not fully understood even though many hypotheses have been proposed [5, 9]. Consequently, a number of human and animal studies have been conducted to elucidate the mechanisms of HAAF. Below, we have reviewed the studies in humans and animals that used different approaches to model the HAAF phenomenon.

## 4. Reproducing the HAAF Phenomenon in Humans

HAAF phenomenon is not specific to diabetes but rather specific to the hypoglycaemia stimulus, as it can be reproduced in healthy individuals; therefore, a number of research groups have attempted to investigate the mechanisms of HAAF in both nondiabetic and diabetic humans. Below, we describe different protocols used for inducing HAAF in humans including insulin dosage, depth, duration and number of antecedent hypoglycaemic episodes, plasma adrenaline responses, and symptom scores. Although the original definition of HAAF phenomenon by Cryer refers specifically to the reduction of neurogenic (autonomic) symptoms rather than neuroglycopenic symptoms [10, 52, 55], both neurogenic and neuroglycopenic symptoms are reduced in response to recurrent hypoglycaemia as evidenced by human studies [14, 15, 18–21]; therefore, both types of symptomatic responses are covered in this review.

### 4.1. General Protocol

Commonly, subjects of average age 26.8 ± 3.6 years with an average body mass index of 24.1 ± 2.3 were chosen for most of the studies [15, 16, 19, 22–27]. Generally, the subjects were fasted overnight prior to all episodes of hypoglycaemia with very few studies fasting the subjects only short-term or with no fasting prior to a subsequent hypoglycaemic episode. All subjects were inserted with two indwelling catheters, one inserted into a hand vein for infusion or injection of insulin, glucose, and drugs and the second placed in the contralateral forearm for blood sampling. The arm for blood sampling was kept in plexiglass box or thermoregulated sleeve and maintained at 55°–65°C to obtain arterialised venous blood samples. Glucose clamps are always used in human studies in order to maintain the blood glucose at the desired level and reduce it in a safe and stepwise manner. It aids in delivering appropriate dosage of insulin with simultaneous infusion of 20% dextrose at a required variable rate. Blood glucose levels were regularly tested at 5 min intervals using either glucose oxidase method, glucose dehydrogenase method, or a handheld device so that the rate of infusion of dextrose could be altered accordingly. Blood samples were collected at regular intervals for measurement of levels of counterregulatory hormones including glucagon, adrenaline, noradrenaline, cortisol, and growth hormone during the euglycaemic (normoglycaemic) and hypoglycaemic episodes. During all euglycaemic and hypoglycaemic episodes, hypoglycaemic symptoms were measured by asking the subjects to score six neurogenic symptoms, that is, (1) heart pounding, (2) shakiness/tremulousness, (3) nervousness/anxiousness, (4) sweating, (5) hunger, and (6) tingling, and six neuroglycopenic symptoms, that is, (1) difficulty in thinking/confusion, (2) tiredness/drowsiness, (3) weakness, (4) feeling warm, (5) fainting, and (6) dizziness [14–27]. Counterregulatory and symptomatic responses to subsequent hypoglycaemia were measured between 18 and 24 hours after the last episode of antecedent hypoglycaemia in majority of the studies [14–19, 22–27].

### 4.2. Diabetic Humans

Diabetic subjects with an average HbA1c level of 7 ± 0.6% were chosen in most of the studies to indicate adequate glycaemic control [15–17]. In addition, since hypoglycaemic events prior to the start of experimentation could confound the counterregulatory and symptomatic responses during studies, the occurrence of such episodes was avoided through (1) frequent monitoring (or self-monitoring) of the blood glucose levels for 1 day [14, 16] or 3 days [15] prior to the start of the study [15, 16] and (2) excluding the patients with a record of hypoglycaemic events two weeks prior to the start of the study. It should be noted that diminished adrenaline response to recurrent hypoglycaemia has been demonstrated for up to 72 hours and 8 days in nondiabetic subjects in two separate studies [21, 56]; therefore, avoiding hypoglycaemic events for 1–3 days used in the above studies may have not been sufficient to ensure the intact counterregulation in diabetic patients at the start of the experiments. The most commonly used dosage of insulin in diabetes patients to cause antecedent and subsequent hypoglycaemia was 1-2 mU/kg/min (see Table 1). The depth of antecedent hypoglycaemic episodes ranged between 2.2 and 3.3 mmol/L and the duration of the episodes was maintained between 1.5 and 2 hours. The number of antecedent hypoglycaemic episodes reported by different laboratories varied from 1 to 4 [14–17]. Studies in type 1 diabetic patients have shown that one or two antecedent hypoglycaemic episodes are sufficient to significantly reduce the plasma adrenaline response and/or symptom responses to a subsequent episode [14–17]. However, another study conducted in type 2 diabetic patients has reported that plasma adrenaline response to recurrent hypoglycaemia was significantly reduced only in the nondiabetic control group but not in insulin treated type 2 diabetes patients [18]. Analysis of plasma adrenaline levels was performed by high-performance liquid chromatography (HPLC) using electrochemical detection [15] or radioimmunoassays [14, 16–18] and reported values were ranging between 187 and 817 pg/mL during antecedent hypoglycaemia and between 75 and 545 pg/mL during subsequent hypoglycaemia (see Table 1 for more details for specific studies). Although the magnitude of the adrenaline response to hypoglycaemia varied between different studies (which could be due to the different degrees of hypoglycaemia achieved and different methodologies to measure adrenaline), most of the studies reported about 40–50% reduction in plasma adrenaline in response to repeated hypoglycaemia in diabetic humans (see Table 1). Both neurogenic and neuroglycopenic symptoms of hypoglycaemia as scored by these patients were also reported to be significantly reduced during subsequent hypoglycaemia in majority of the studies that measured the symptoms [14, 15, 18].

Overall, these data suggest that the HAAF phenomenon has mostly been successfully reproduced in diabetic humans. The major challenge in investigating the HAAF phenomenon in diabetic patients, however, is the possible occurrence of unidentified hypoglycaemic episodes prior to the beginning of the study, which is a significant limitation. Another challenge is that the glycaemic threshold in diabetic subjects might have been reset to a lower blood glucose level due to previous recurrent hypoglycaemic episodes [57, 58]. Thus the counterregulatory responses and hypoglycaemia symptoms will only be triggered at much lower blood glucose levels, which may not be ethically achievable. Hence, investigation of HAAF in nondiabetic humans, without profound effects of complications associated with diabetes or prior unidentified hypoglycaemic episodes, may be a valuable alternative. Studies that investigated the HAAF phenomenon in healthy individuals are covered below.

### 4.3. Nondiabetic Humans

Following the study by Heller and Cryer [19] which successfully demonstrated the HAAF phenomenon in nondiabetic individuals, researchers have tried to reproduce the phenomenon in nondiabetic subjects to study the mechanisms of HAAF.

Nondiabetic subjects for the studies were chosen based on their general wellbeing with no family history of diabetes. The dosage of insulin to induce antecedent and subsequent hypoglycaemia in healthy individuals in most studies ranged from 1 to 2 mU/kg/min (see Table 1). The number of antecedent hypoglycaemic episodes in most studies was 2, which generally produced a 40–50% reduction in plasma adrenaline following subsequent hypoglycaemia (see Table 1). However, Moheet et al. [21] reported that a significant reduction in plasma adrenaline and symptom scores is only achievable with 3 antecedent hypoglycaemic episodes, not 2. The depth of hypoglycaemia during antecedent episodes ranged from 2.2 to 3.3 mmol/L. Davis et al. [59] tested the effects of different depths of antecedent hypoglycaemia on counterregulatory responses in nondiabetic humans and reported that when the blood glucose level was reduced to 3.9 mmol/L during antecedent episodes only plasma adrenaline and glucagon responses to subsequent hypoglycaemia are significantly reduced whereas blood glucose levels of 3.3 mmol/L or lower during antecedent hypoglycaemia lead to a significant reduction in glucagon, adrenaline, noradrenaline, and growth hormone responses to subsequent hypoglycaemia. The duration of antecedent hypoglycaemic episodes varied between 40 min and 3 hours in different studies. Davis et al. [60] have compared the effects of short-term (5 min), intermittent (30 min), and prolonged (90 min) antecedent hypoglycaemic episodes on the counterregulatory and symptomatic responses to subsequent hypoglycaemia. They reported that the plasma adrenaline response was similar irrespective of the duration of antecedent hypoglycaemia but hypoglycaemia symptoms responses were only reduced when the antecedent episodes were either intermittent or prolonged. Thus the depth and duration of antecedent hypoglycaemia must be carefully considered to achieve a significant reduction in both plasma adrenaline response and symptom scores.

Plasma adrenaline responses to antecedent and subsequent hypoglycaemia were measured by HPLC with electrochemical detection or by radioimmunoassay. The levels of plasma adrenaline reported by different groups varied greatly (which, again, could be due to the different degrees of hypoglycaemia achieved and different methodologies to measure adrenaline); however, most studies have reported a significant reduction in plasma adrenaline from 300–950 pg/mL during antecedent hypoglycaemia to 150–450 pg/mL during subsequent hypoglycaemia (see Table 1).

Some studies measured neuroglycopenic and neurogenic symptom scores during antecedent and subsequent hypoglycaemic episodes. They generally reported that the symptoms were significantly reduced during subsequent hypoglycaemia, while a reduction in neuroglycopenic but not neurogenic symptom score was reported by Klement et al. [24] and no significant reduction in symptom scores occurred in a study by Goldberg et al. [22]. These inconsistences may arise from the variability of symptoms and age differences (18–49 years old) between participants in the Goldberg et al. [22] study.

Most of the studies tested the counterregulatory and symptomatic responses to subsequent hypoglycaemia 18–24 hours after the last episode of antecedent hypoglycaemia [15–17, 19, 22]. Moheet et al. [21] have induced two episodes of antecedent hypoglycaemia on day 1 and one episode on day 2 and achieved a significant reduction in both plasma adrenaline and symptom scores in response to subsequent hypoglycaemia on day 5. George et al. [56] have induced one episode of antecedent hypoglycaemia on day 1 and compared the counterregulatory and symptom responses to consequent hypoglycaemia on days 3 and 8 and reported that on day 8 the symptom scores were already restored to normal whereas the sympathoadrenal responses were still diminished. Hence, the time of measurement of the counterregulatory and symptomatic responses to subsequent hypoglycaemia after the last episode of antecedent hypoglycaemia is critical for successful reproduction of the HAAF phenomenon as the responses to hypoglycaemia could be restored to normal after a specific period.

Taken together, most of the studies have successfully reproduced the HAAF in healthy individuals when two antecedent hypoglycaemic episodes lasting for about 90 min (with blood glucose levels reduced to and maintained at 3 mmol/L) were induced on day 1 followed by the third hypoglycaemic episode on day 2.

## 5. Reproducing the HAAF Phenomenon in Rodents

Rodents are widely used to investigate the mechanisms of HAAF and have many advantages compared to studying the phenomenon in humans. Animal models provide the opportunity to achieve lower blood glucose levels facilitating the counterregulatory responses to be studied at much lower glycaemic threshold which is not possible in humans for ethical reasons. There is also the possibility of investigating the changes at the tissue levels apart from the measurement of blood glucose and plasma hormone levels. Animal models are the only option when invasive procedures are required, for example, to investigate changes at the central nervous system level. Animal models are also useful to examine the efficacy of possible drugs to restore the plasma adrenaline response. A challenge in using animal models to study the HAAF phenomenon is the inability to measure hypoglycaemic symptom responses, an important component of the phenomenon. Furthermore, some of the hormonal and tissue responses to hypoglycaemia may differ in rodents and thus pharmacological approaches might vary from humans. For example, in nondiabetic humans and mice, glucagon response to subsequent hypoglycaemia is reported to be significantly reduced [14–17, 19, 20, 22–25, 27, 47, 48] whereas studies in nondiabetic rats are inconclusive, with some studies reporting a significant reduction in glucagon responses to subsequent hypoglycaemia [28–31] and others reporting no significant difference [32–36].

Using rodents to model the HAAF phenomenon has been challenging as many factors can influence its reproducibility including insulin dosage, route of insulin administration, depth and duration of hypoglycaemia, and number of antecedent hypoglycaemic episodes. Most of the studies have used Sprague-Dawley rats and some have used Wistar rats (see Table 2). From the bulk of literature, it is noticeable that research groups have used different approaches to modelling the HAAF phenomenon in rodents and the results published in the literature are quite variable. Below we summarise the recent findings and also describe our own experiences in modelling HAAF in rats.

### 5.1. Nondiabetic Rats

#### 5.1.1. Route of Insulin Administration

**Figure 3:**
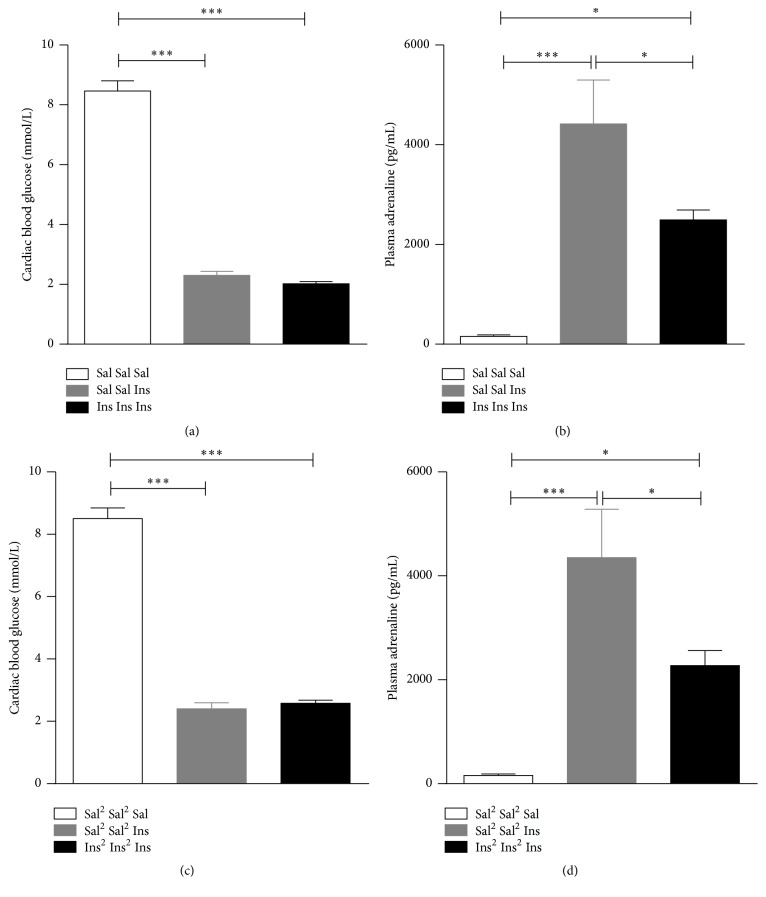
Cardiac blood glucose (a, c) and plasma adrenaline (b, d) levels in rats exposed to saline (white bars), single hypoglycaemia (grey bars), or recurrent hypoglycaemia (black bars). In the recurrent hypoglycaemia groups, rats were exposed to one (a, b) or two episodes (c, d) of antecedent hypoglycaemia per day for two consecutive days followed by an additional episode of hypoglycaemia on day 3. Sal or Sal^2^: saline given once or twice a day, respectively; Ins or Ins^2^: insulin given once or twice a day, respectively. Data are presented as mean ± SEM. ^*∗*^ *p* < 0.05; ^*∗∗∗*^ *p* < 0.001.

There are three routes by which insulin is administered in rats: (1) subcutaneous (s.c), (2) intraperitoneal (i.p), and (3) intravenous (i.v). Some studies have adopted s.c route for insulin administration to produce antecedent [37] or for both antecedent and subsequent hypoglycaemic episodes [28, 29, 32]. However, subcutaneous administration of insulin reportedly produced variable depth and length of hypoglycaemia when compared to i.p administration [33]. A number of studies have performed i.p administration of insulin to induce antecedent hypoglycaemia [30, 33, 34, 38, 61]. Although the most preferred route of administration in many studies is the i.v route [35, 36, 39, 40, 42–44] (see Table 2), some researchers have used a combination of i.p and i.v or s.c and i.v with the i.p or s.c injections used to induce antecedent hypoglycaemia and the i.v administration or continuous i.v infusion used to produce the subsequent hypoglycaemic episode [30, 31, 33, 34, 37, 41]. Intravenous infusions have the advantage of achieving the desired hypoglycaemia levels relatively quickly, but it requires the animals to be surgically fitted with venous catheters to enable researchers to perform repeated i.v administration and thus requires specialised skills which could be challenging. In our laboratory, we have adopted the i.p method of insulin administration to induce single [62] or recurrent hypoglycaemia (see Figure 3) and observed that this route successfully reduced the blood glucose levels consistently throughout our experiments.

#### 5.1.2. Insulin Dose

The insulin dose used to induce antecedent and subsequent hypoglycaemic episodes was widely different across all studies that have attempted to establish the HAAF phenomenon in rodents (see Table 2).

A number of studies using rodents have successfully reproduced the phenomenon by administering 1–10 U/kg insulin to induce both antecedent and subsequent hypoglycaemia [28, 32–37, 40–43, 61] or to induce antecedent hypoglycaemia and then used a continuous infusion of insulin at a rate of 20–50 mU/kg/min to induce subsequent hypoglycaemia [30, 33, 34, 38, 41, 61]. It must be noted that high plasma insulin levels as used in some of these studies may not be relevant to the human condition. Also, administration of high insulin concentrations may not necessarily reproduce the phenomenon more successfully. For example, Figlewicz et al. [44] and Paranjape and Briski [29] who administered 6.75 U/kg and 12 U/kg insulin, respectively, to induce both antecedent and subsequent hypoglycaemia were unable to achieve a significant reduction in plasma adrenaline response to subsequent hypoglycaemic episode. However, these two studies have not reported the levels of hypoglycaemia achieved with these insulin concentrations. Osundiji et al. [36] administered insulin doses which were progressively reduced from a starting dose of 10 U to 6–8 U/kg, between days 1 and 4, arguably to account for the increased sensitivity to repeated insulin treatments. In contrast, Evans et al. [43] used a higher dose of insulin on the second day to produce subsequent hypoglycaemia, presumably to overcome insulin resistance caused by repeated insulin injections [63]. In our own studies we have found that daily 10 U/kg insulin injections to nonfasted rats on 3 consecutive days produce a milder hypoglycaemia on day 4 (3 mmol/L) than on day 1 (2 mmol/L) (unpublished observation), suggesting that repeated high dose insulin injections may cause insulin resistance in rats. Since other factors such as route of administration, fasting conditions, depth, duration, and number of antecedent episodes (as highlighted in this manuscript) can also alter the plasma adrenaline response to subsequent hypoglycaemia, insulin dosage has to be carefully established in conjunction with these other factors to achieve the phenomenon successfully.

#### 5.1.3. Fasting Conditions

As in the case of insulin dose, different research groups have used different protocols for fasting animals prior to treatments. Some studies have not fasted the animals [28, 29, 36, 37, 39–44], whereas others have overnight fasted the animals only prior to the induction of the subsequent hypoglycaemia [30, 34, 35, 38]. Orban et al. [32] fasted their animals for only an hour before the injection to induce antecedent hypoglycaemic episodes. In our laboratory, we overnight fasted the rats only before the last hypoglycaemic episode.

#### 5.1.4. Blood Sampling Methods

Blood glucose levels are generally measured either from the tail or from the systemic circulation with the use of the vascular catheters. Blood glucose levels measured from the tail vein can be misleading at times regarding the depth of hypoglycaemia as the tail vein blood glucose is generally lower than that of the systemic circulation. In our experiments we noticed this discrepancy between the tail vein and the cardiac blood glucose. The cardiac blood glucose level was consistently higher than the tail vein blood glucose levels in all our rats (8 mmol/L and 6 mmol/L, resp.). Hence the depth of hypoglycaemia if measured from the tail vein should be used in conjunction with the observation made from the wellbeing of the animals, for example, if the animals are responsive, are able to move, and grip when lifted. The method of analysis of blood glucose levels was also variable between studies. Most of the studies used glucose oxidase method whereas some of the studies used handheld devices to measure blood glucose levels (see Table 2).

Most studies collected blood samples for hormone analyses from catheters at regular intervals [30, 33–35, 37–44, 61]. In our laboratory, blood samples for adrenaline analysis were collected through cardiac puncture at 60 min from the time of injection during antecedent and subsequent hypoglycaemia in different sets of animals. The advantage of using cardiac blood in rats for hormonal analysis is that it allows obtaining large quantities of plasma (up to 4-5 mL) without inducing hypovolemic stress; thus many analyses can be performed on the same blood sample.

#### 5.1.5. Depth and Duration of Hypoglycaemia

The depth of hypoglycaemia achieved in some studies ranged between 2 and 3.2 mmol/L [30, 32–34, 36, 38, 40, 44] during antecedent hypoglycaemic episodes whereas in other studies blood glucose levels have been lowered below 2 mmol/L [28, 35, 37, 39, 42, 43, 61] which may not be relevant to the human condition. During subsequent hypoglycaemic episodes some studies clamped the blood glucose levels at an average of 2.5 mmol/L to avoid excessive hypoglycaemia. This clamping is achieved by regular monitoring of tail vein or systemic blood glucose with corresponding amount of dextrose infused. Duration of antecedent hypoglycaemic episodes is also highly variable between research groups, ranging from 1 hour to 3 hours. Figlewicz et al. [44] have not reported on the depth of hypoglycaemia during antecedent hypoglycaemia and kept the duration of antecedent hypoglycaemic episode for only an hour and Paranjape and Briski [29] have not measured blood glucose levels during antecedent hypoglycaemic episodes and the duration of the episodes is not reported. It is possible these are the reasons for not achieving a significant reduction in the plasma adrenaline levels in response to subsequent hypoglycaemia observed in these two studies and thus implies the importance of depth and duration of antecedent hypoglycaemic episodes. In our laboratory, food was removed for 120 min from the time of injection during antecedent hypoglycaemic episodes and blood glucose levels were measured at 30 min intervals during this time until the 120 min time point. This protocol lowered the blood glucose level below 3 mmol/L and produced a significant reduction in plasma adrenaline response to subsequent hypoglycaemia after one antecedent hypoglycaemia episode per day for two days (see Figure 3).

#### 5.1.6. Number of Antecedent Hypoglycaemia Episodes

In humans, it has been shown that even a single episode of hypoglycaemia can significantly diminish counterregulatory responses to the subsequent hypoglycaemia. However, in rats, one to five antecedent hypoglycaemic episodes have been induced to achieve a significant reduction in plasma adrenaline response to subsequent hypoglycaemia. Flanagan et al. [33] have shown diminished plasma adrenaline response to subsequent hypoglycaemia 48 hours after a single antecedent hypoglycaemia. Some researchers have shown diminished response to acute hypoglycaemia after 2 episodes of antecedent hypoglycaemia on the previous day [28, 42–44] whereas some have induced 1 episode of antecedent hypoglycaemia per day for 2 to 4 days [32–35, 37, 39–41, 61] prior to testing the response to subsequent hypoglycaemia (see Table 2). Kudrick et al. [38] have tested 1 and 2 antecedent hypoglycaemic episodes a day and reported that the plasma adrenaline response to subsequent hypoglycaemia was significantly reduced only in the rats that had two hypoglycaemic episodes a day, which is contradictory to a number of the abovementioned studies that have successfully achieved a significant reduction in plasma adrenaline response with 1 episode of antecedent hypoglycaemia for 1, 2, or 3 days. In our laboratory, we tested the effects of one or two episodes of antecedent hypoglycaemic episodes per day for two consecutive days on the adrenaline secretion in response to subsequent hypoglycaemia on day 3 (Figure 3). All of our experiments involving animals were approved by the SA Pathology Animal Ethics Committee (Australia). Adult male Sprague-Dawley rats were handled for 2 weeks prior to the experiments and were subsequently injected with 10 U/kg insulin (Ins) or saline (Sal) i.p once a day for three days (Ins Ins Ins; Sal Sal Sal) or twice a day on two consecutive days and once on the third day (Ins^2^ Ins^2^ Ins; Sal^2^ Sal^2^ Sal). Two more sets of animals received saline once or twice on the first two days and insulin once on the third day to represent the single hypoglycaemia groups (Sal Sal Ins; Sal^2^ Sal^2^ Ins). Animals were overnight fasted before the last injection and were euthanized 60 min from the time of last injection. Blood sampling for glucose was performed using a handheld glucometer and plasma adrenaline measurements were carried out using an ELISA assay as described by us previously [62]. One-way ANOVAs followed by Tukey's test for multiple comparisons were used to assess the effect of treatment between groups using PRISM V6.05 (GraphPad Software, Inc., United States of America).

In our experiment, we did not observe any significant difference in the plasma adrenaline response to subsequent hypoglycaemia between the one and two episodes per day protocols (Figure 3) with both protocols causing about a 50% reduction in the plasma adrenaline response to subsequent hypoglycaemia when compared to the respective acute hypoglycaemia groups (Sal Sal Ins and Sal^2^ Sal^2^ Ins, resp.) that achieved similar levels of hypoglycaemia.

Thus it seems a single episode of antecedent hypoglycaemia per day for 1–3 days or 2 episodes per day for 1 or 2 days can lead to a significant reduction in plasma adrenaline response to subsequent hypoglycaemia in nondiabetic rats.

#### 5.1.7. Plasma Adrenaline Responses

A significant reduction in plasma adrenaline response to subsequent hypoglycaemia when compared to that of a single episode of hypoglycaemia marks the successful reproduction of the HAAF phenomenon. Analysis of plasma adrenaline was generally performed by HPLC using electrochemical detection [32–35, 37, 39–41], ELISA [30, 36, 38], or radioenzymatic assay [28, 42–44, 61]. Even though most of the studies have reported a significant reduction in plasma adrenaline response to subsequent hypoglycaemia, the levels of plasma adrenaline reported are highly variable between studies. For example, Al-Noori et al. [42] reported that plasma adrenaline levels after a single hypoglycaemia episode were 4080 pg/mL that reduced after the subsequent episode to 2358 pg/mL; however, Osundiji et al. [36] reported 450 pg/mL of plasma adrenaline in response to single episode of hypoglycaemia which was reduced to approximately 175 pg/mL after 4 episodes of hypoglycaemia. Nevertheless, most of the studies have successfully achieved a significant reduction in plasma adrenaline response to subsequent hypoglycaemia despite using different protocols with different insulin dosage, fasting conditions, number of antecedent hypoglycaemic episodes, and depth and duration of antecedent hypoglycaemic episodes.

From our observations, insulin administered intraperitoneally consistently produced a significant reduction in blood glucose levels below 3 mmol/L within 30 min and stayed low until 120 min in nonfasted animals during antecedent hypoglycaemic episodes. Also one such hypoglycaemic episode per day for two days significantly reduced the plasma adrenaline response at 60 min from the time of injection during the subsequent hypoglycaemia on day 3 (Figure 3).

### 5.2. Diabetic Rats

Very few research groups have attempted to reproduce the HAAF phenomenon in streptozotocin-induced diabetic rats (see Table 2). McNay and Sherwin [45] have administered 6–10 U/kg insulin i.p once a day for 3 days that lowered the blood glucose levels to 2.8 mmol/L. This protocol has produced a significant reduction in the plasma adrenaline response to subsequent hypoglycaemia on day 4. However, there was no difference in plasma adrenaline response to subsequent hypoglycaemia in another study in which 2 U/kg insulin was administered subcutaneously to induce 2 episodes of antecedent hypoglycaemia per day for 3 days and one episode on the 4th day that lowered the blood glucose levels to 2.2 mmol/L [46]. This could be due to variations in the counterregulatory responses arising due to complications of diabetes when compared to that of nondiabetic rats. A challenge faced in reproducing this phenomenon in diabetic rats appears to be the need for a higher dosage of insulin to overcome insulin resistance and the altered counterregulatory response due to the diabetes itself. Another challenge could be requiring significantly more animals as the criteria to be classified as diabetic is not met by all of the rats after streptozotocin treatment and the process of inducing diabetes itself can be challenging and more demanding. Nevertheless, as the HAAF phenomenon is the major problem in diabetic patients, it is important that researchers continue investigating this phenomenon in both diabetic and nondiabetic animals to better understand its mechanisms and the influence of diabetes.

### 5.3. Nondiabetic Mice

Reproducing the HAAF phenomenon in nondiabetic mice has been attempted only by two research groups (see Table 2). In a study by Jacobson et al. [47], antecedent hypoglycaemia with continuous infusion of insulin at 20 mU/kg/min failed to reduce the plasma adrenaline significantly during subsequent episode. Additionally, i.p administration of 2.5 U/kg insulin that lowered blood glucose levels to 2.2 mmol/L once a day for 3 days actually increased the plasma adrenaline response to subsequent hypoglycaemia, rather than reducing it [48]. Thus the HAAF phenomenon is yet to be successfully reproduced in nondiabetic mice.

## 6. Conclusions and Future Directions

Overall, both human and animal models of HAAF are extremely valuable in exploring the possible mechanisms of the phenomenon despite their limitations. However, care needs to be taken to consider the species used to model the phenomenon, route of administration, insulin dosage, fasting conditions, depth, duration, and number of antecedent hypoglycaemic episodes to ensure successful reproduction of the phenomenon. The important points to consider for future research in modelling the HAAF phenomenon are as follows: (1) avoiding hypoglycaemic events in diabetic patients and animals prior to experimentation is important to ensure that counterregulatory and symptomatic responses to hypoglycaemia are intact; (2) human studies in diabetic patients should report what selection/exclusion criteria were used for recruitment of participants to demonstrate that their plasma adrenaline and hypoglycaemia symptoms were not altered prior to experimentation; (3) animal studies should avoid using excessively high concentrations of insulin resulting in severe hypoglycaemia, as such conditions are not representative of the human phenomenon; (4) most animal studies have been performed in nondiabetic animals; more studies in diabetic animals are needed to establish if the HAAF phenomenon can be reproduced in rodents with diabetes similar to humans; (5) future research is needed to investigate the duration of diminished adrenaline release and impaired symptoms after antecedent hypoglycaemia in response to subsequent hypoglycaemia in both nondiabetic and diabetic patients and animals; this information will be critical for successful modelling of the HAAF phenomenon in the future.

## Figures and Tables

**Table 1:** Summary of procedures used to reproduce the HAAF phenomenon in diabetic and nondiabetic humans.

Subjects	Fasting condition	Insulin dose used to induce antecedent hypoglycaemia	Insulin dose used to induce subsequent hypoglycaemia	Depth and duration of antecedent hypoglycaemia	Method of glucose assay	Number of antecedent episodes	Plasma adrenaline in response to antecedent/subsequent hypoglycaemia (in pg/mL) and method of assay	Symptom scores during subsequent hypoglycaemia relative to that of antecedent hypoglycaemia
Type 1 diabetes patients [14–17]	Overnight fasted prior to all days of experiments [16, 17] Overnight fasted prior to subsequent episode [14, 15]	1-2 mU/kg/min [14–17]	1-2 mU/kg/min [14–17]	2.2–3.3 mmol/L [14–17] 1.5–2 hrs [14, 16, 17]	Glucose dehydrogenase method [15] Glucose oxidase method [14, 16, 17]	1/day for 1 day [14, 16] 2/day for 1 day [17] 1/day for 4 days [15]	380/200; *p* = 0.0060 [14] 187/75; *p* < 0.05 [17] 817/545; *p* < 0.05 [15] 300/200; *p* = 0.0397 [16] HPLC [15] RIA [14, 16, 17]	Reduced neurogenic symptoms and neuroglycopenic symptoms *p* < 0.05–0.001 [14, 15] Not measured [17] No significant change [16]

Type 2 diabetes patients [18]	Overnight fasted prior to all days of experiments [18]	1-2 mU/kg/min [18]	1-2 mU/kg/min [18]	2.2–3.3 mmol/L [18] 1.5–2 hrs [18]	Glucose oxidase method [18]	2/day for 1 day [18]	No significant change in insulin treated patients *p* = 0.07 [18] RIA [18]	Reduced neurogenic symptoms and neuroglycopenic symptoms *p* < 0.05–0.001 [18]

Nondiabetic subjects [19–27]	Nonfasted [24] Overnight fasted prior to all days of experiments [19–25, 27]	1-2 mU/kg/min [19–27]	1-2 mU/kg/min [19–27]	2.2–2.8 mmol/L [21–24] 2.9–3.3 mmol/L [19, 20, 23, 25, 27] 40 min–2 hrs [20, 21, 23–26] 3 hrs [22]	Glucose oxidase method [20–23, 25, 27] HemoCue handheld device [24, 26]	1/day for 1 day [19] 2/day for 1 day followed by 1/day on day 2 [21] 2/day for 1 day [22–27] 1/day for 1 day followed by interval interprandial hypoglycaemia [20]	300–500/150–250; *p* < 0.05–0.001 [20–22, 25] 800–1000/350–500; *p* < 0.05–0.01 [19, 23, 26, 27] No significant change [24] HPLC [21, 22, 24, 26, 27] RIA [20, 23]	Not measured [23, 25, 27] No significant change [22] Reduced neuroglycopenic symptoms *p* < 0.04; no significant change in neurogenic symptoms [24] Reduced neurogenic symptoms and neuroglycopenic symptoms; *p* < 0.05–0.001 [19–21]

RIA: radioimmunoassay; HPLC: high-performance liquid chromatography.

**Table 2:** Summary of procedures used to reproduce the HAAF phenomenon in rodents.

	Species	Route of administration	Insulin dose used to induce antecedent hypoglycaemia	Insulin dose used to induce subsequent hypoglycaemia	Fasting condition	Blood glucose measurement (method of sampling and assay)	Depth and duration of antecedent hypoglycaemia	Number of antecedent hypoglycaemic episodes	Plasma adrenaline in response to antecedent/subsequent hypoglycaemia (in pg/mL) and method of assay
Nondiabetic rats	Sprague-Dawley rats [28–41] Wistar rats [42–44]	s.c [28, 29, 32] i.v [35, 36, 39, 40, 42–44] s.c and i.v [37] i.p and i.v [30, 31, 33, 34, 41]	1–3 U/kg [28, 30, 32, 37, 38, 40, 42, 43] 5–10 U/kg [31, 33, 34, 39, 41, 44] 12 U/kg [29] 4 U/kg and additional 0.5–1 U/kg [35] 10 U/kg and 6–8 U/kg [36]	1–3 U/kg [28, 40, 42] 5–10 U/kg [28, 35, 36, 43, 44] 12 U/kg [29] 20–50 mU/kg/min [30, 31, 33, 34, 38, 41] 0.75 U/kg followed by 1.8 U/kg/hr [37] 0.75 U/kg 3 times at 5 min interval [35]	Fasted overnight [33] Nonfasted [28, 29, 31, 36, 37, 39–44] Overnight prior to subsequent hypoglycaemia [30, 34, 35, 38] Fasted for four hours prior to subsequent hypoglycaemia [32]	Tail [32, 34, 40] Systemic [28, 30, 35, 39, 41–44] Tail/systemic [31, 36–38] Trunk [29] Glucometer [30, 32, 36, 40] Glucose oxidase method [28, 29, 33–39, 42, 43] Modified Trinder's reaction assay [44]	1.4–1.9 mmol/L [28, 31, 35, 37, 39, 42, 43] 2–3.2 mmol/L [30, 32–34, 36, 38, 40] 1-2 hrs [28, 31, 32, 35, 37, 39, 40, 42–44] 3 hrs [30, 33, 34, 36, 38, 41] Depth and duration not given [29]	1/day for 1 day [33, 35, 39] 1/day for 2 days [37] 1/day for 3-4 days [29, 31, 32, 34, 36, 40, 41] 2/day for 1 day [28, 42–44] 2/day for 2 days and 1/day on third day [30, 38]	450–1000/175–460; *p* < 0.05–0.01 [28, 32, 36] 1000–3000/740–1500; *p* < 0.05–0.01 [30, 37–40, 43] 3000–5000/1500–2400; *p* < 0.05–0.01 [31, 33–35, 41, 42] 1600/1000; *p* > 0.05 [44] 3800/3250; *p* > 0.05 [29] HPLC [31–35, 37, 39–41] RIA [28, 42–44] ELISA [29, 30, 36, 38]

Diabetic rats	Streptozotocin-induced diabetic Sprague-Dawley rats [45, 46]	i.p [45] s.c and i.v [46]	6–10 U/kg [45] 2 U/kg [46]	6–10 U/kg [45] 10 mU/kg/min [46]	Nonfasted [45] Overnight prior to subsequent hypoglycaemia [46]	Not given [45] Systemic [46] Glucose oxidase method [45, 46]	2.8 mmol/L [45] 2.2 mmol/L [46] 3 hrs [45] 1 hr [46]	1/day for 3 days [45] 2/day for 3 days and 1/day on 4th day [46]	3010/890; *p* < 0.05 [45] 5076/5076; *p* > 0.05 [46] HPLC [45] RIA [46]

Nondiabetic mice	C57BL/6 [47, 48]	i.p [48] i.v [47]	2.5 U/kg [48] 20 mU/kg/min [47]	2.5 U/kg [48] 20 mU/kg/min [47]	3 hours prior to injections on all days [48] 6 hours prior to each episode [47]	Tail [48] Systemic [47] Glucometer [47, 48]	2.2 mmol/L [48] 3.8 mmol/L [47] 3 hrs [48] 2 hrs [47]	1/day for 4 days [48] 1/day for 1 day [47]	14000/15000; *p* > 0.05 [48] 220/120; *p* > 0.05 [47] HPLC [47] ELISA [48]

RIA: radioimmunoassay; HPLC: high-performance liquid chromatography; ELISA: enzyme linked immunosorbent assay.
